# Doxorubicin induces trans-differentiation and MMP1 expression in cardiac fibroblasts via cell death-independent pathways

**DOI:** 10.1371/journal.pone.0221940

**Published:** 2019-09-12

**Authors:** Masatoshi Narikawa, Masanari Umemura, Ryo Tanaka, Mayu Hikichi, Akane Nagasako, Takayuki Fujita, Utako Yokoyama, Tomoaki Ishigami, Kazuo Kimura, Kouichi Tamura, Yoshihiro Ishikawa

**Affiliations:** 1 Cardiovascular Research Institute, Yokohama City University School of Medicine, Yokohama, Japan; 2 Medical Science and Cardiorenal Medicine, Yokohama City University School of Medicine, Yokohama, Japan; 3 Department of Physiology, Tokyo Medical University Graduate School of Medicine, Tokyo, Japan; National Institutes of Health, UNITED STATES

## Abstract

Although doxorubicin (DOX)-induced cardiomyopathy causes lethal heart failure (HF), no early detection or effective treatment methods are available. The principal mechanisms of cardiotoxicity are considered to involve oxidative stress and apoptosis of cardiomyocytes. However, the effect of DOX on cardiac fibroblasts at non-lethal concentrations remains unknown. The aim of this study was to investigate the direct effect of doxorubicin on the activation of cardiac fibroblasts independent of cell death pathways. We first found that DOX induced α-SMA expression (marker of trans-differentiation) at a low concentration range, which did not inhibit cell viability. DOX also increased MMP1, IL-6, TGF-β and collagen expression in human cardiac fibroblasts (HCFs). In addition, DOX promoted Akt and Smad phosphorylation. A Smad inhibitor prevented DOX-induced α-SMA and IL-6 protein expression. An PI3K inhibitor also prevented MMP1 mRNA expression in HCFs. These findings suggest that DOX directly induces fibrotic changes in HCFs via cell death-independent pathways. Furthermore, we confirmed that these responses are organ- and species-specific for HCFs based on experiments using different types of human and murine fibroblast cell lines. These results suggest potentially new mechanisms of DOX-induced cardiotoxicity from the viewpoint of fibrotic changes in cardiac fibroblasts.

## Introduction

Associated with the remarkable developments in cancer treatment and the aging of patients with cancer, anticancer drugs have increased mortality due to adverse effects [1]. Cardiac complications are the most lethal side effects, and thus, the cardiotoxicity of anticancer drugs has recently received much attention [2]. Because doxorubicin (DOX) causes a high incidence of congestive heart failure (HF), many mechanisms of DOX-induced cardiotoxicity have been reported [3]. DOX-induced cardiotoxicity is associated with impairment of several cellular functions in cardiomyocytes, including generation of reactive oxygen species (ROS) [4], mitochondrial dysfunction [5], and inhibition of topoisomerase2β [6], leading to cellular oxidative stress and activation of various cell death pathways, such as apoptosis and necrosis pathways. Nevertheless, effective prophylaxis and early detection approaches for DOX-induced cardiotoxicity have not been developed [7].

As many as 70% of all myocardial cells are non-myocyte cells, including cardiac fibroblasts (CFs), endothelial cells and vascular smooth muscle cells [8]. Among them, fibroblasts are involved in production of extracellular matrix (ECM) proteins [9] and humoral factors, such as transforming growth factor-β (TGF-β), interleukin-6 (IL-6) and platelet-derived growth factor (PDGF). In response to various stressors, CFs trans-differentiate into myofibroblasts, which synthesize larger amounts of ECM and gain contractile activity due to expression of α-smooth muscle actin (α-SMA) [10] [11]. However, few reports have demonstrated the direct effect of DOX on CFs. Zhan et al. showed that Ataxia telangiectasia mutated in murine CFs regulates DOX-induced cardiotoxicity [12]. Gosh et al. found that an inhibitor of PAI-1 effectively prevents DOX-induced senescence in cardiomyocytes, fibroblasts and endothelial cells [13]. Moreover, almost all the in vitro studies that have examined DOX-induced cardiotoxicity performed experiments with more than 1 μM DOX. However, the onset and threshold of DOX-induced cardiotoxicity are still unclear. Therefore, we have attempted to reveal the biomarker for early diagnosis or the potential risk of DOX-induced cardiotoxicity. In this study, we focused on the direct response of CFs exposed to low-dose DOX because we focused on the importance of the effect of DOX on cellular function apart from cell death.

IL-6 levels are highly correlated with the severity of heart failure, myocardial infarction and cardiac fibrosis [14] [15]. CFs are the major source of IL-6, compared with cardiomyocytes and endothelial cells, in heart tissue [16]. IL-6 induces the development of inflammation and cardiac remodeling [17]. We also reported that TGF-β induced IL-6 production and hyperthermia treatment inhibited this regulation in human cardiac fibroblasts (HCFs) [18]. In addition, matrix metalloproteinases (MMPs) regulate cardiac fibrosis via degradation of ECM [19]. Especially, MMP1 can digest type-1 and type-3 collagens, which are key components of the ECM in cardiac tissue. Previously, we revealed that hydrostatic pressure inhibited IL6, α-SMA and MMP1 mRNA expression in HCFs [20]. Many reports have demonstrated a high correlation between MMP1 expression and heart failure [19] [21].

Here, we examined the direct effects of DOX on CFs and investigated the associated signaling mechanism. We show that low-dose DOX induced IL-6, α-SMA, MMP1 and collagen expression in HCFs, most likely via PI3K/Akt and TGFβ/Smad signaling. Our findings support the existence of new mechanisms by which DOX can induce cardiac fibrosis via upregulation of inflammatory cytokines and MMP1, resulting in trans-differentiation of HCFs.

## Materials and methods

### Reagents

Reagents were purchased from Sigma-Aldrich (MO, USA) unless otherwise specified. The antibodies used in western blotting are described in each section. Doxorubicin hydrochloride was purchased from Sigma-Aldrich (MO, USA). LY294002 (PI3K inhibitor) and SB431542 (TGF-β/Smad inhibitor) were purchased from Cell Signaling (MA, USA).

### Cell culture and DOX stimulation

Human cardiac fibroblasts (HCFs) were purchased from ScienCell Research Laboratories (CA, USA) and maintained in FM-2 medium supplemented with 1% penicillin-streptomycin (PS), 1% FGS-2 and 2% fetal bovine serum (FBS) [22]. Normal human dermal fibroblasts (NHDFs) were purchased from Lonza (Basel, Switzerland) and maintained in Dulbecco’s modified Eagle’s medium (DMEM) (10% FBS). Murine cardiac fibroblasts (mCFs) were isolated from 8-week-old adult mice using a modified procedure previously described [23]. Briefly, mouse hearts were isolated under sterile conditions and kept in Hank’s balanced salt solution (HBSS). After being washed with fresh HBSS, the hearts were transferred into DMEM with 1% penicillin-streptomycin supplemented with 0.1% trypsin, 0.5 mg/ml collagenase type II (Worthington Biochemical Corporation, NJ, USA) and 0.01 mM HEPES. The hearts were then minced into 1-mm^3^ pieces, transferred into 50-mL centrifuge tubes, and incubated on an orbital shaker at 37°C for 20 min with the collagenase solution as described above. After ice-cold HBSS was added into the digest to inhibit collagenase activity, dispersed cells were separated from non-digested tissues by passage through a 40-μm-diameter cell strainer and collected via centrifugation. After being washed once by centrifugation in HBSS, the cells were resuspended in 10 mL DMEM supplemented with 10% FBS and 1% PS and seeded into sterile culture dishes. mCFs were also cultured in DMEM (10% FBS), and passage 1 cells were used in the microarray analysis. All cells were maintained in a humidified 5% CO_2_ atmosphere at 37°C. In general, HCFs from passages 4–8 were used. Prior to treatment, culture medium was replaced with DMEM (5% FBS, 1% FGS-2). Following appropriate time of stimulation, cell lysates and mRNA were collected as will be described later. To measure the expression of ACTA2 and MMP1 mRNA, we stimulated cells with DOX (0.1μM) for 24 hours. To measure the expression of IL-6 and TGF-β mRNA, cells were exposed to DOX (0.1μM) for 3 to 6 hours. To examined the expression of collagen mRNA, cells were exposed to DOX (0.1μM) for 24 to 72 hours. To evaluate the time dependency of protein expression of α-SMA, HCFs were exposed to DOX (0.1μM) and total cell lysate was collected after 24, 48 and 72 hours. To examine the dose dependency of protein expression of α-SMA and MMP1, cells were exposed to DOX (0.005 to 0.1 μM) for 48 hours. To examine the phosphorylation of some signaling protein, we stimulated HCFs with DOX (0.1μM) and total cell lysate was collected after 10 to 60 minutes. In the experiments using pharmacological inhibitors, LY294002 (10 μM) or SB431532 (1 μM) was added to the cells, 30 minutes prior to DOX treatment. Untreated cells cultured under similar conditions served as controls. Culture medium supernatant was collected, and MMP1 concentration was determined with an enzyme-linked immunosorbent assay (ELISA).

### Microarray

The effects of DOX on mRNA expression profiles were evaluated by microarray analysis. HCFs, NHDFs and mCFs were exposed to 0.1 μM DOX for 6 hours or 24 hours, and then, total RNA was extracted from each cell type. Microarray experiments were carried out using a SurePrint G3 Human GE 8x60K v3 Microarray and a SurePrint G3 Mouse Gene Expression 8x60K v2 Microarray (Agilent Technologies, CA, USA) according to the manufacturer’s protocol, with total RNA as the starting material.

### Cell viability assay

Cell proliferation assays were performed with a commercial 2,3-bis(2-methoxy-4-nitro-5-sulfophenyl)-5-[(phenylamino)carbonyl]-2H-tetrazolium inner salt (XTT) Cell Proliferation Assay Kit (ATCC, VA, USA) as previously reported [18]. We stimulated HCFs with DOX (0.1 to 0.5 μM) for 48 hours or 72 hours, and cell viability was measured with an XTT assay kit.

### Western blotting

Western blot analyses were performed as previously described [18]. Briefly, cells were lysed and sonicated in RIPA buffer (Thermo Scientific, IL, USA). Equal amounts of protein were subjected to sodium dodecyl sulfate polyacrylamide gel electrophoresis (SDS-PAGE). After electrophoretic separation, protein bands were transferred to a Millipore Immobilon-P membrane followed by immunoblotting with antibodies against molecules of interest. The following primary antibodies were used for immunoblotting: anti-Smad2 (Cell Signaling, MA, USA, 1:1000), anti-p-Smad2 (Cell Signaling, 1:1000), anti-Smad3 (Cell Signaling, 1:1000), anti-p-Smad3 (Cell Signaling, 1:1000), anti-Akt (Cell Signaling, 1:1000), anti-p-Akt (Thr308 and Ser473) (Cell Signaling, 1:1000), anti-S6K (Cell Signaling, 1:1000), anti-p-S6K (Cell Signaling, 1:1000), anti-α-SMA (SIGMA, 1:2000), anti-fibronectin (Abcam, 1:1000), anti-vinculin (SIGMA, 1:1000), anti-paxillin (BD Bioscience, 1:1000), anti-MMP1 (Santa Cruz Biotechnology, CA, USA, 1:1000) and anti-GAPDH (Santa Cruz Biotechnology, 1:5000) antibodies. Chemiluminescence detection was performed using ECL reagent (Bio-Rad Laboratories. Inc., CA, USA). The signal intensities of bands were quantified with ImageJ software (NIH).

### Quantitative real-time reverse transcriptase-polymerase chain reaction (RT-PCR)

Isolation of total RNA, generation of cDNA and RT-PCR analysis were performed as previously described [18]. The sequences of the specific primers were as follows: IL-6 (forward, 5′- CCAGGAGCCCAGCTATGAA -3′; reverse, 5′-TTCTGCCAGTGCCTC TTTG -3′), TGF-β (forward, 5′-TACCTGAACCCGTGTTG CTC -3′; reverse, 5′-CCGGTAGTGAACCCGTTGAT -3′), ACTA2 (forward, 5′- ATTGCCGACCGAA TGCAGA -3′; reverse, 5′- ATGGAGCCACCGATCC AGAC -3′), MMP1 (forward, 5′-CTCTGGAGTAATGTCACACCTCT -3′; reverse, 5′-TGTTGGTCCACCTTTCATC TTC -3′), col1A1 (forward, 5′- CCCGGGTTTCAGAG ACAACTTC -3′; reverse, 5′- TCCACATGCTTTATTCCAGCAATC -3′), MMP-2 (forward, 5’- GATACCCCTTTGA CGGTAAGGA -3’; reverse, 5’- CCTTCTCCCAAGG TCCATAGC -3’), MMP-9 (forward, 5’- TGTACCGCTATGGTTACACTCG-3’; reverse, 5’- GGCAGGGACAGTTGCTTCT-3’) and 18S (forward, 5′- GTAACCCGTTGAACCCCATT-3′; reverse, 5′- CCATCCAATCGGTAG TAGCG-3′). PCR consisted of an initial cycle of 95°C for 30 sec and then 40 cycles, each consisting of denaturation at 95°C for 5 sec, followed by annealing and primer extension at 60°C for 30 sec. Melting curve analysis was conducted from 60°C to 95°C, with a heating rate of 0.3°C per sec. The abundance of each gene was determined relative to that of the 18S transcript.

### Measurement of IL-6 and MMP1 via enzyme-linked immunosorbent assay (ELISA)

The secretion of IL-6 was measured using a human IL-6 quantitative ELISA kit (R&D Systems Inc., MN, USA) according to the manufacturer’s instructions. The production of MMP1 in the presence of DOX for 48 h was measured in HCFs using a human MMP1 quantitative ELISA kit (RayBiotech, GA, USA) and the activation of MMP1 was measured using MMP-1 assay kit (ANASPEC, CA, USA) according to the manufacturer’s instructions.

### Data analysis and statistics

Values represent the mean ± SEM. Statistical comparisons among groups were performed using Student’s *t*-test, one-factor analysis of variance (ANOVA) or two-way ANOVA with a Bonferroni post hoc test. The criterion of statistical significance was set as *p*<0.05. Significant differences are indicated by *p<0.05, **p<0.01, and ***p<0.001; ns, not significant.

## Results

### DOX markedly induced cytokine expression in human cardiac fibroblasts and normal human dermal fibroblasts compared with murine cardiac fibroblasts

**Fig 1:**
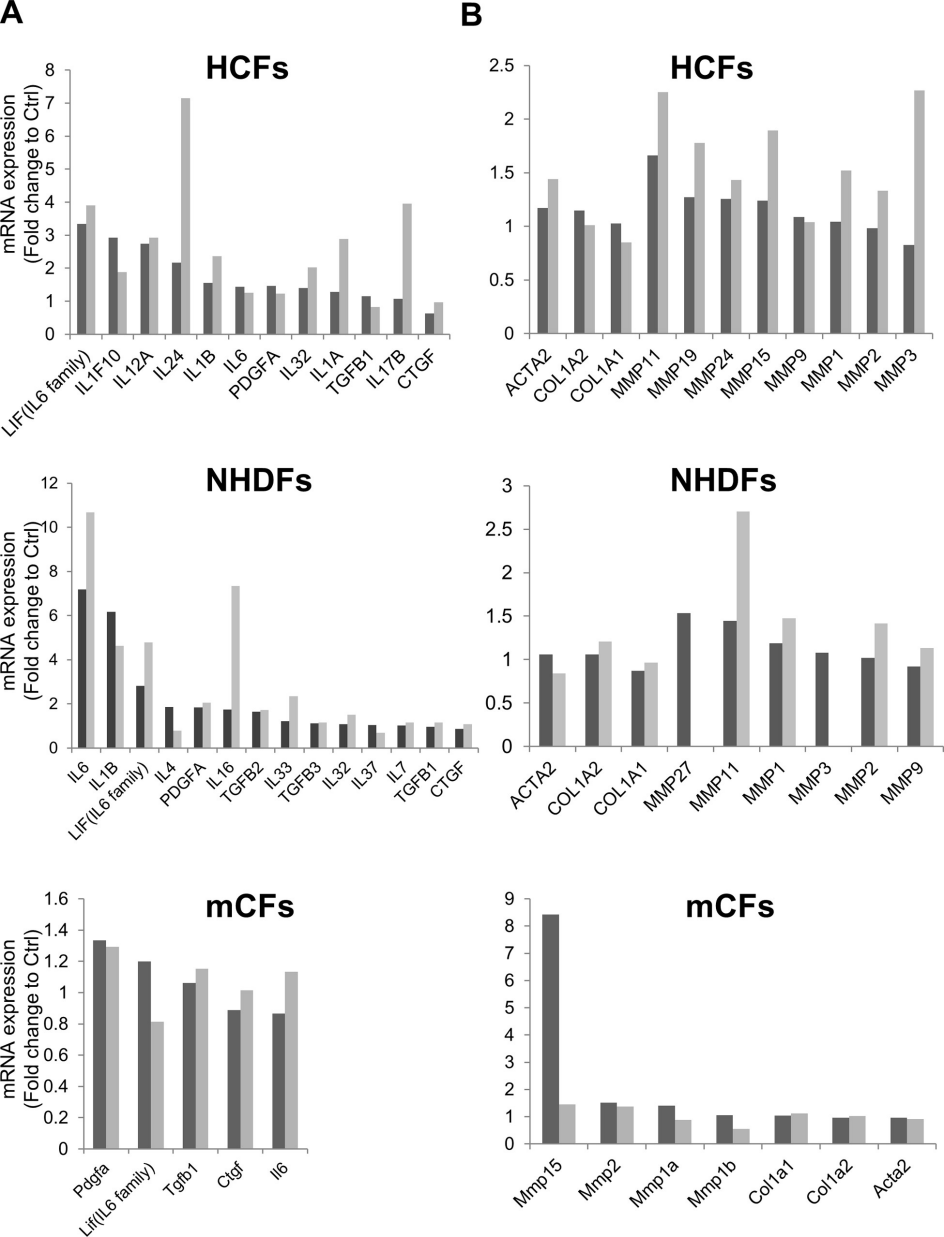
Microarray analysis of gene expression induced by DOX in HCFs, NHDFs and mCFs. Human cardiac fibroblasts (HCFs), normal human dermal fibroblasts (NHDFs) and murine cardiac fibroblasts (mCFs) were exposed to 0.1 μM DOX, and mRNA expression levels were analyzed with a microarray. A, Cytokine-associated gene expression in HCFs, NHDFs and mCFs at 6 (black) and 24 (gray) hours. B, Fibrosis-associated and MMP gene expression in HCFs, NHDFs and mCFs at 6 (black) and 24 (gray) hours. (n = 1).

We first performed a cell viability assay to examine the cytotoxicity of DOX toward HCFs. An XTT assay showed that DOX did not decrease cell viability at less than 0.1 μM (S1 Fig). We next performed a microarray analysis to investigate the effect of DOX on gene expression in HCFs. Furthermore, we compared the expression pattern in HCFs with that in normal human dermal fibroblasts (NHDFs) and murine cardiac fibroblasts (mCFs) under DOX stimulation. Microarray analysis showed that DOX increased the expression of certain cytokines in HCFs and NHDFs but had little effect on mCFs (Fig 1A). DOX increased leukemia inhibitory factor (LIF), (an IL-6 family cytokine), IL-1, IL-17 and IL-24 expression in HCFs. DOX also increased LIF, IL-1, IL-6 and IL-16 expression in NHDFs. Meanwhile, DOX did not significantly change fibrotic gene expression in HCFs and NHDFs after 6 and 24 hours (Fig 1B). With regard to MMP genes, which are related to cardiac remodeling, DOX also increased MMP1 and MMP2 expression in HCFs and NHDFs. According to these results, we focused on IL-6 expression at the early phase in HCFs and NHDFs under DOX stimulation and MMP1 and MMP2 expression at the late phase in HCFs and NHDFs exposed to DOX. Furthermore, we evaluated certain fibrotic markers (*i*.*e*., α-SMA and collagen) at 24 hours and onward.

### DOX induced trans-differentiation of human cardiac fibroblasts but not human dermal fibroblasts

**Fig 2:**
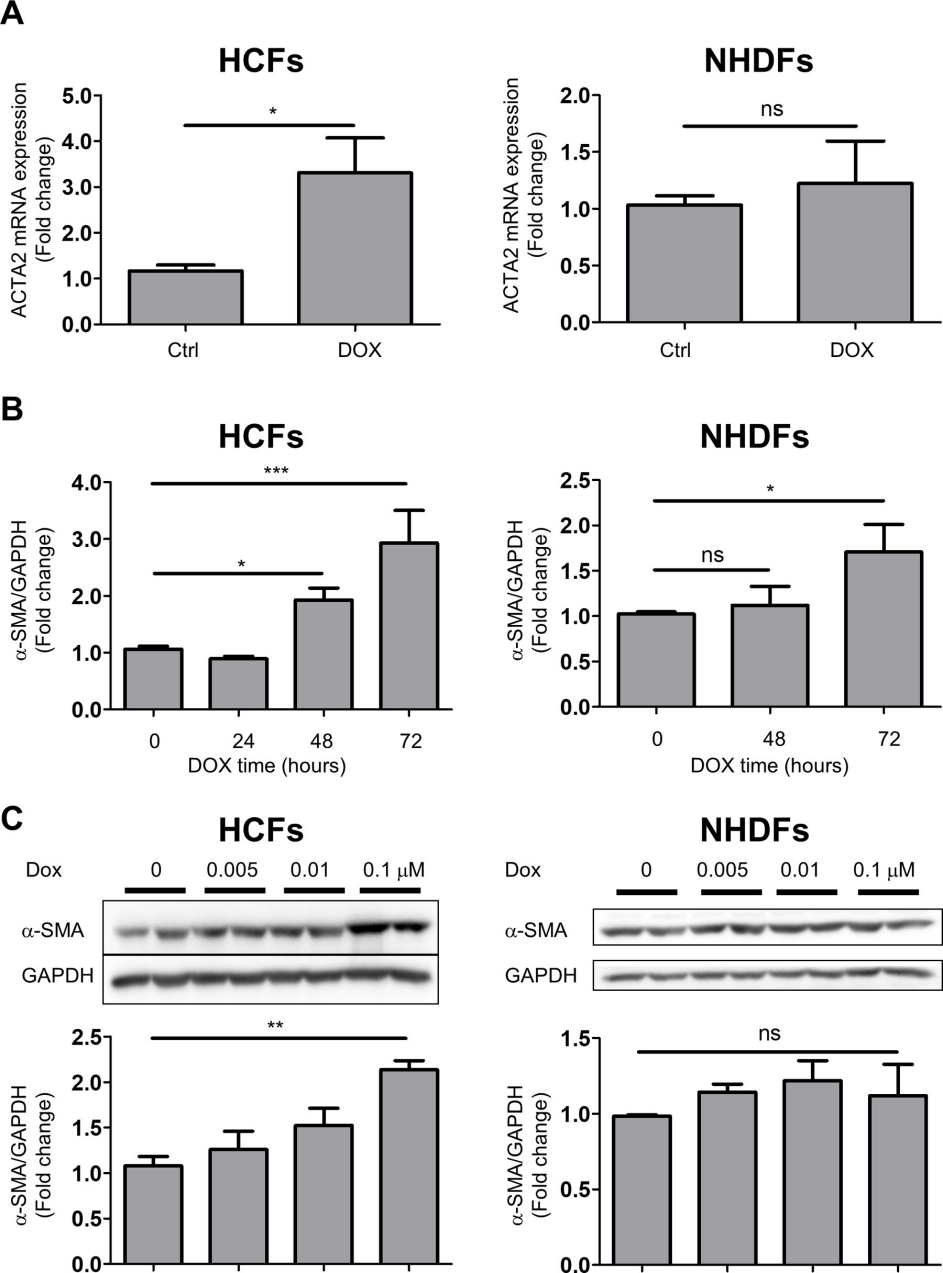
DOX induced trans-differentiation of HCFs but not NHDFs. A, ACTA2 mRNA expression in the presence of DOX for 24 hours in HCFs (left) and NHDFs (right) (n = 5–6; *p < 0.05; ns: no significant difference). B, α-SMA protein expression in the presence of DOX (0.1 μM) for 24 to 72 hours in HCFs (left) and NHDFs (right) (n = 4–6; **p < 0.01, and *p < 0.05). C, α-SMA protein expression in the presence of DOX (0.005 to 0.1 μM) for 48 hours in HCFs (left) and NHDFs (right) (n = 4; **p < 0.01; ns: no significant difference). The original immunoblot image is shown in S2 Fig.

HCFs can trans-differentiate into myofibroblasts and induce a fibrotic response in injured myocardium [10]. The defining marker of differentiated myofibroblasts is the expression of αα-SMA [24]. To investigate the effect of DOX on trans-differentiation of HCFs, we evaluated α-SMA expression via RT-PCR and western blot analysis. RT-PCR showed that DOX significantly increased ACTA2 mRNA (which codes for α-SMA) expression after 24 hours (Fig 2A). In contrast, the ACTA2 mRNA level was not significantly changed in NHDFs. Moreover, DOX increased α-SMA protein expression in a time- and a dose-dependent manner (Fig 2B and 2C). In contrast, DOX did not increase α-SMA protein expression in NHDFs for 48 hours (Fig 2B). To confirm the potential trans-differentiation ability of NHDFs, we stimulated NHDFs with TGF-β. TGF-β significantly increased α-SMA protein expression after 24 hours in NHDFs (S3 Fig). To present the supporting evidence about trans-differentiation of HCFs, we examined the expression of the other molecular markers. DOX upregulated the expression of fibronectin, vinculin and paxillin in only HCFs (S4 Fig). These findings suggest that DOX-induced trans-differentiation was a specific response in cardiac fibroblast cells.

### DOX increased activity of MMP1 in human cardiac fibroblasts via regulation of mRNA and protein expression

**Fig 3:**
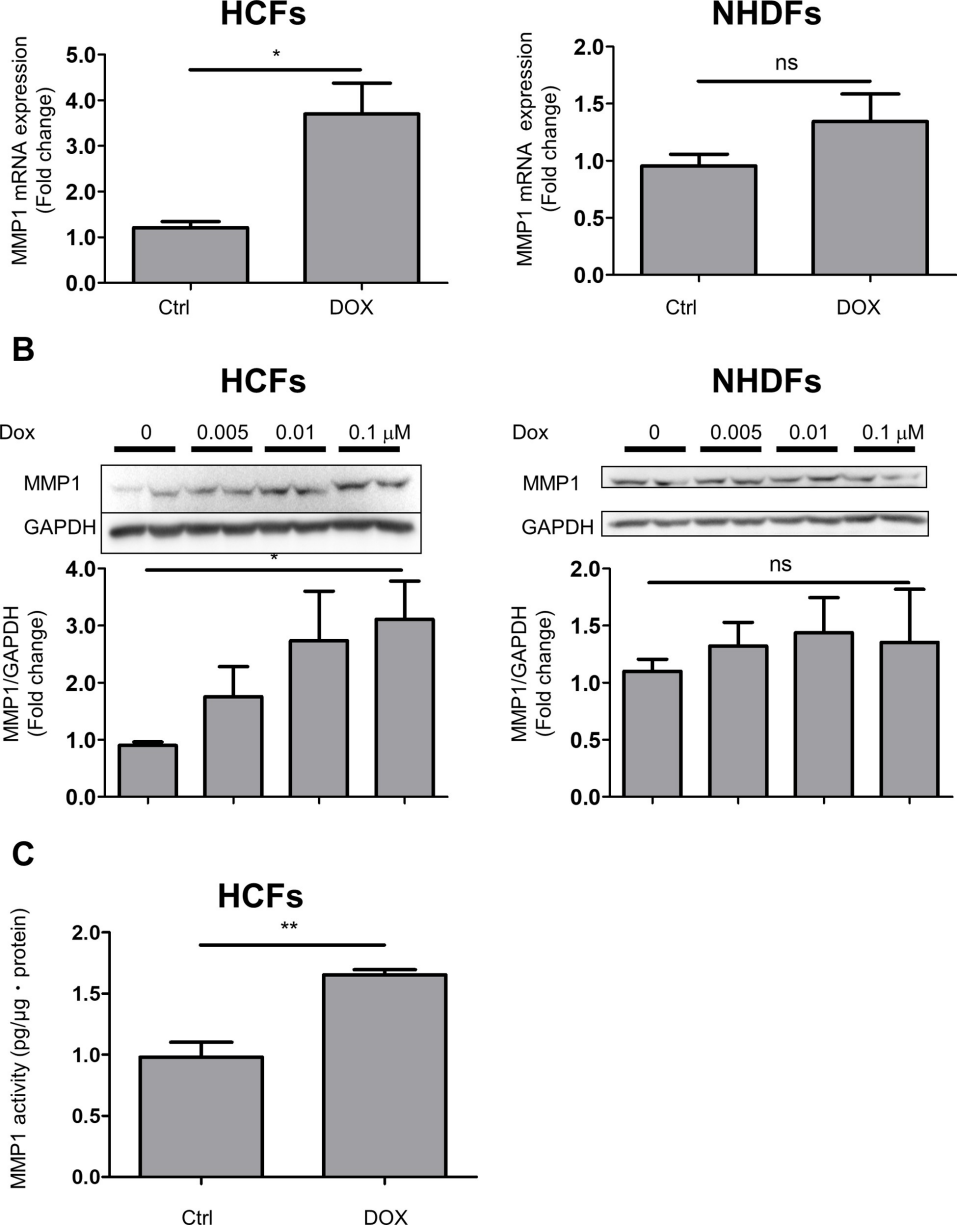
DOX induced MMP1 expression and production in HCFs but not in NHDFs. A, MMP1 mRNA expression in the presence of DOX (0.1 μM) for 24 hours in HCFs (left) and NHDFs (right) (n = 4–5; *p < 0.05; ns: no significant difference). B, MMP1 protein expression in HCFs (left) and NHDFs (right) stimulated by DOX (0.1 μM) for 48 hours (n = 4–6; *p < 0.05; ns: no significant difference). The original immunoblot image is shown in S2B Fig. C, Activity of MMP1 in supernatant from HCFs exposed to DOX (0.1 μM) for 48 hours (n = 4; **p<0.01).

Because MMPs have an ability to degrade ECM in cardiac tissue, MMPs are known as key regulators of cardiac fibrosis [19]. We examined MMP expression in HCFs exposed to DOX. RT-PCR showed that DOX significantly increased MMP1 mRNA levels (Fig 3A), although DOX did not increase MMP2 and MMP9 mRNA expression in HCFs (S5 Fig). Compared with HCFs, DOX did not significantly increase MMP1 mRNA in NHDFs (Fig 3A). We also confirmed that DOX only induced MMP1 protein expression in a dose dependent manner in HCFs (Fig 3B). Moreover, DOX increased MMP1 secretion in HCFs (S6 Fig). In the ELISA, we collected supernatant at 48 hours because there was no significant difference between the control and the DOX group at 24 hours. Finally, we measured activity of MMP1 secreted from HCFs. DOX enhanced activity of MMP1 in HCFs (Fig 3C). These data showed that DOX increased expression of mRNA, secretion and activity of MMP1 in HCFs.

### DOX increased IL-6, TGF-β and collagen mRNA levels in human cardiac fibroblasts

**Fig 4:**
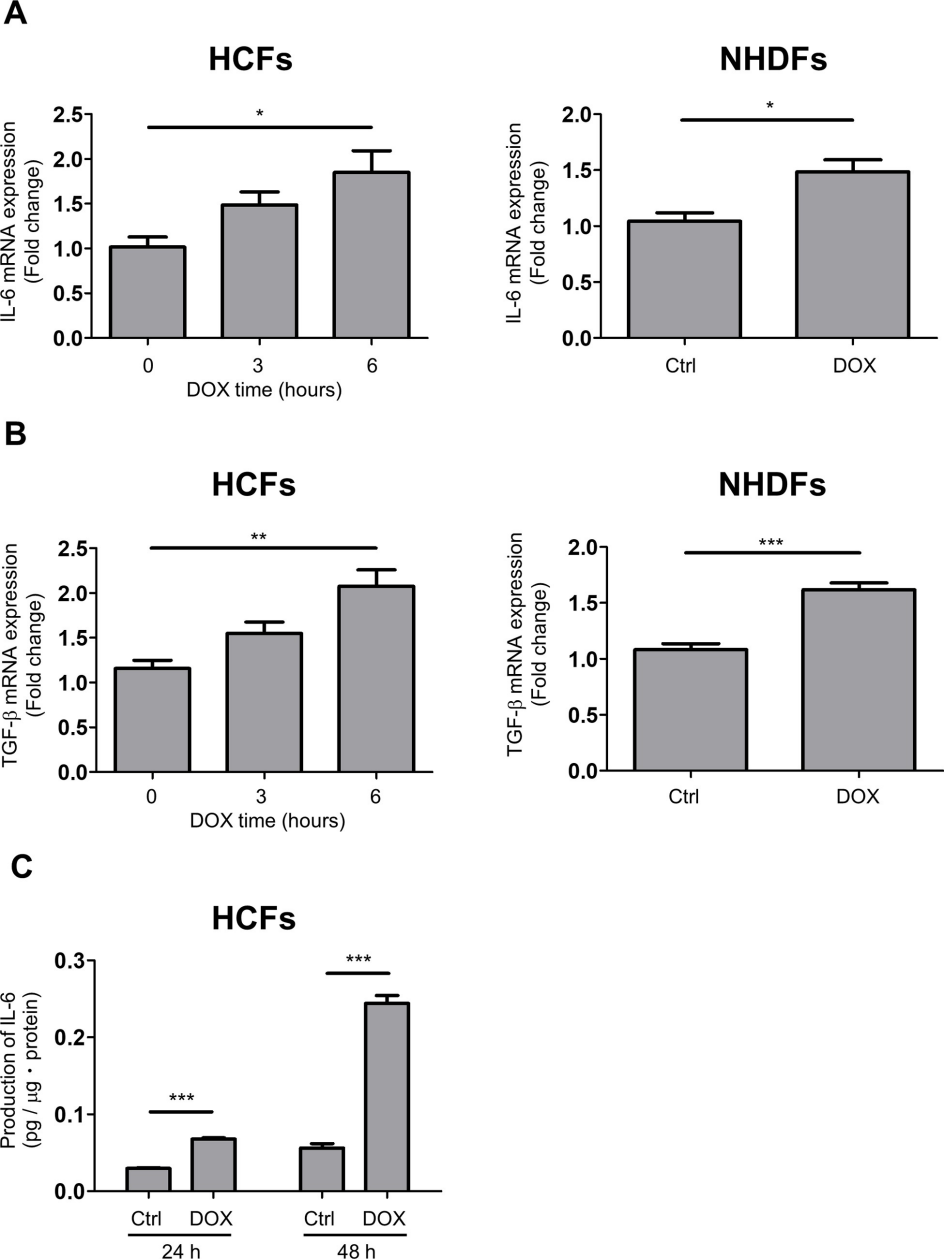
DOX induced IL-6, TGF-β and collagen expression in HCFs. A, IL-6 mRNA expression in HCFs with or without DOX treatment (0.1 μM) for 3 to 6 hours (n = 4; *p < 0.05). B, TGF-β mRNA expression in HCFs with or without DOX treatment (0.1 μM) for 3 to 6 hours (n = 4; **p<0.01, ***p<0.001). C, Production of IL-6 in supernatant from HCFs with DOX treatment (0.1 μM) for 24 to 48 hours (n = 4; ***p<0.001).

One of the important functions of HCFs is the production of growth factors and cytokines in heart tissue. Thus, we measured IL-6 and TGF-β mRNA levels in HCFs exposed to DOX. After 3 and 6 hours of stimulation, DOX increased the mRNA level of IL-6 and TGF-β in a time-dependent manner (Fig 4A and 4B). In contrast, DOX slightly increased the mRNA expression of IL-6 and TGF-β in NHDFs. We also found that DOX increased production of IL-6 in supernatant from HCFs (Fig 4C). Moreover, DOX increased the expression of col1a1 mRNA over 72 hours in HCFs but not in NHDFs (S7 Fig). These results suggest that DOX stimulated inflammatory cytokine expression at the early phase and the production of collagen at the late phase in HCFs.

### DOX activated PI3K/Akt/S6K and Smad signaling in human cardiac fibroblasts

**Fig 5:**
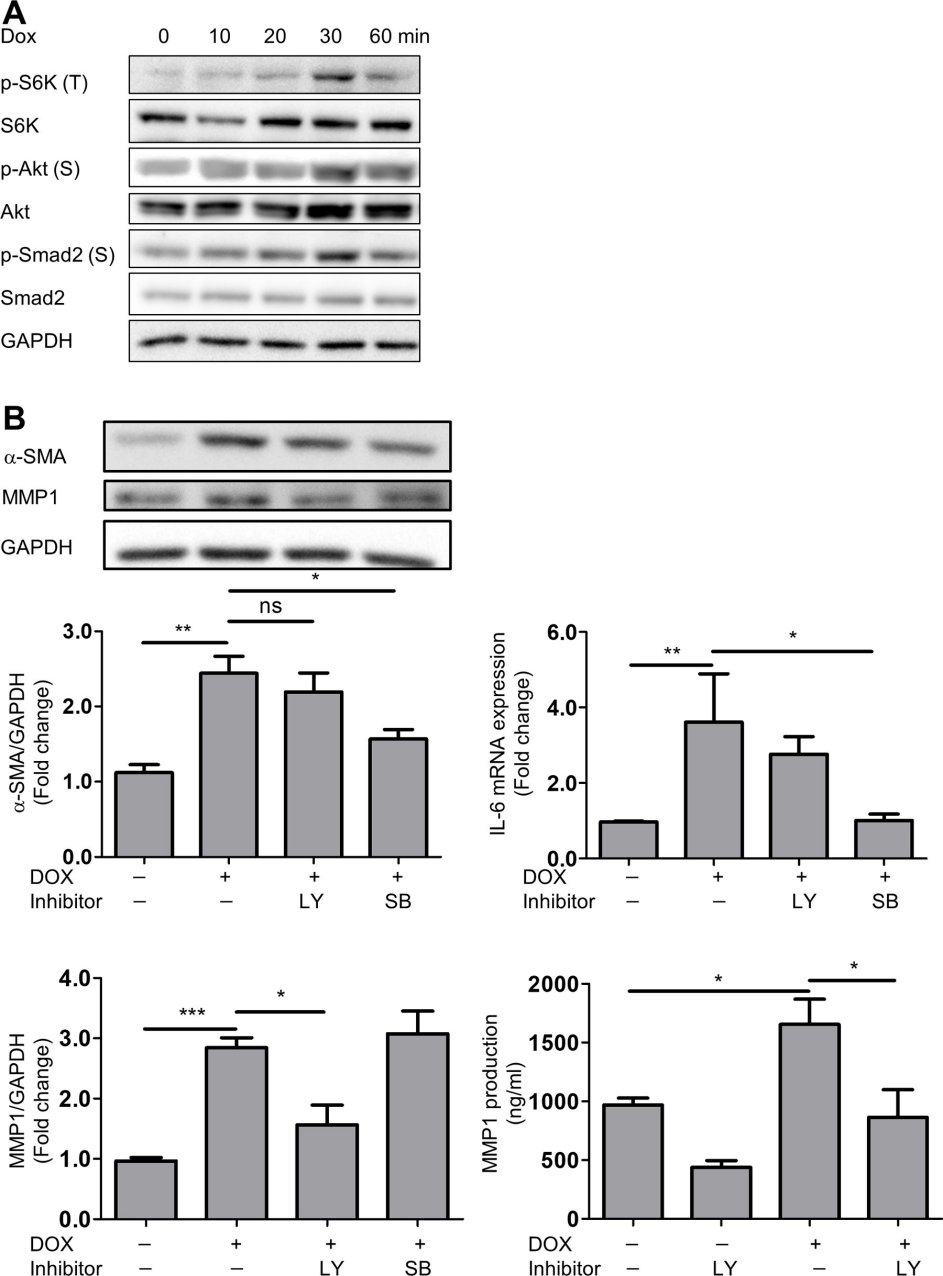
DOX phosphorylated Smad and Akt in HCFs. A, The time course of protein phosphorylation (p-S6K-t389, p-Akt-s473 and p-Smad2-s465/467) in HCFs stimulated by DOX (0.1 μM) for 0 to 60 min. The original immunoblot image is shown in S8 Fig. B, A TGF-β/Smad inhibitor (SB431512) decreased the DOX-induced IL-6 and α-SMA expression. A PI3K inhibitor (LY294002) decreased DOX-induced protein expression and production of MMP1 in HCFs (n = 4–6; *p < 0.05, **p < 0.01, ***p<0.001). The original immunoblot image is shown in S9 Fig.

**Fig 6:**
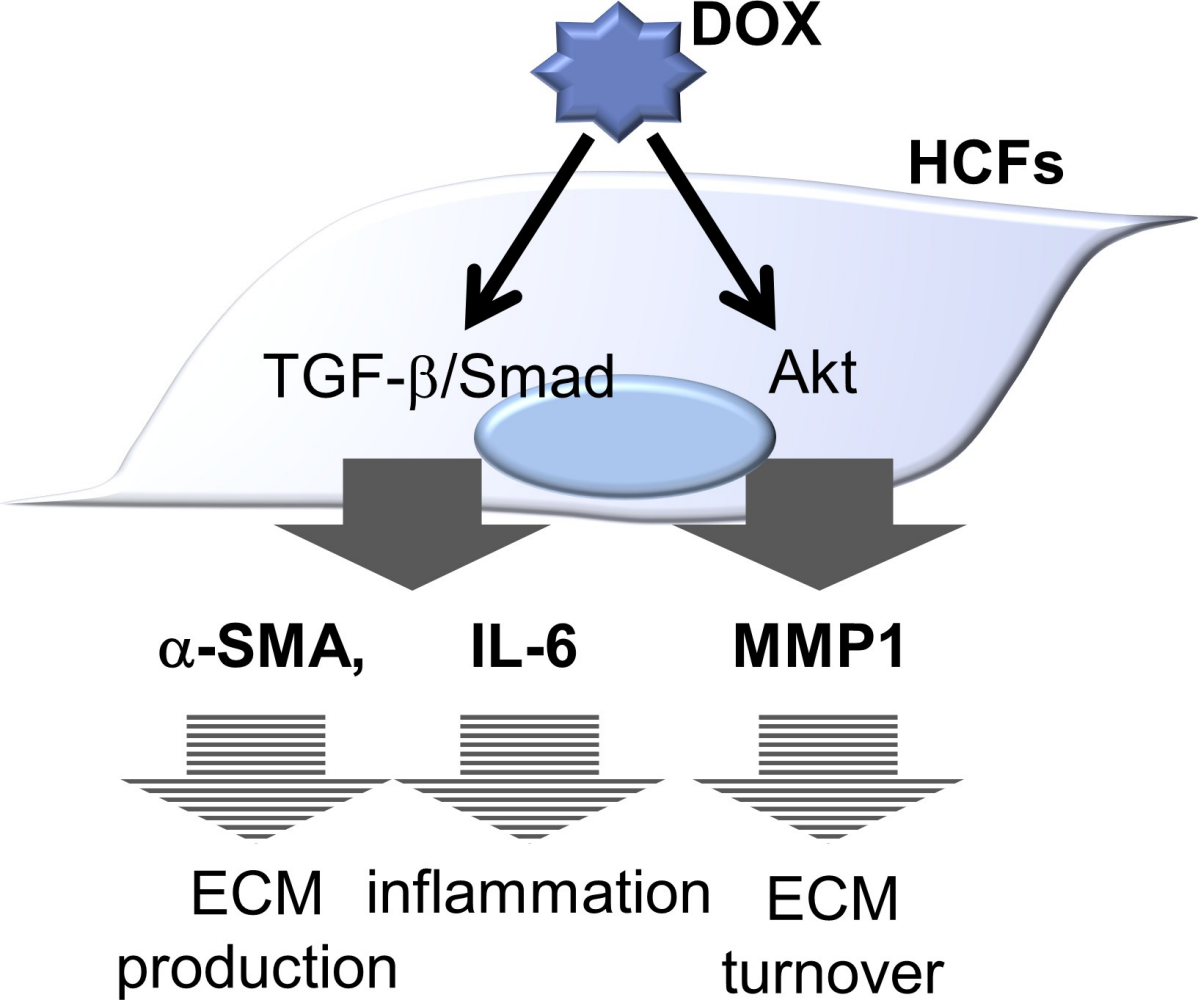
A proposed schematic diagram of DOX-induced pro-fibrotic changes in HCFs. DOX activated Smad and Akt signaling and increased IL-6, α-SMA and MMP1 expression in HCFs.

TGF-β/Smad signaling is well known as a signaling cascade involved in the fibrotic response [25]. In addition, the participation of certain canonical pathways (*i*.*e*., PI3K/Akt, FAK and JNK) in fibroblasts has been reported [26]. We next examined these cellular signaling pathways in HCFs exposed to DOX. We found that DOX upregulated Smad2 and Akt phosphorylation in a time-dependent manner (Fig 5A). To investigate the role of TGF-β/Smad and PI3K/Akt signaling in HCFs stimulated by DOX, we examined whether an PI3K or Smad2 inhibitor prevented DOX-induced IL-6, α-SMA and MMP1 protein expression. Indeed, a TGF-β/Smad inhibitor decreased the DOX-induced IL-6 mRNA and α-SMA protein expression. Moreover, a PI3K inhibitor suppressed the protein expression of MMP1 in HCFs (Fig 5B). These results suggest that DOX induces inflammatory and fibrotic responses via the PI3K/Akt and TGF-β/Smad signaling pathways, which are independent of the other pathways illustrated in Fig 6.

## Discussion

In this study, we demonstrated that DOX induced trans-differentiation of HCFs and production of MMP1 at a non-lethal dose via the TGF-β/Smad and PI3K/Akt signaling pathways. DOX also increased IL-6 expression at the early phase and collagen expression at the late phase in HCFs. Compared with NHDFs, these responses were specific to HCFs.

We first performed a microarray assay to identify the cell-specific response to DOX exposure in HCFs, NHDFs and mCFs. The results shown in Fig 1 reveal that HCFs and NHDFs were highly susceptible to DOX compared with mCFs. Furthermore, Souders et al. mentioned that the exact cellular makeup of the heart can vary dramatically from species to species. These differences in cardiac cell populations can also lead to differences in ECM content [8]. We assumed that our results were caused by differences in species and passage number. Comparing HCFs with NHDFs, DOX increased inflammatory cytokine expression, such as IL-1 and IL-6 family cytokines, in both cell types. The expression of fibrotic factors, such as TGF-β and PDGF, was not markedly changed. Moreover, the mRNA expression of MMP1, MMP2 and MMP11 was upregulated in both cell types. Although MMP1 and MMP2 are well-known regulators of cardiac ECM components [27], MMP11 has been observed in endometrium and human carcinomas [28]. Therefore, we focused on the increase in MMP1 and MMP2 expression.

To compare the cellular response between HCFs and NHDFs in detail, we investigated whether the response is organ-specific. As shown in Fig 2B, DOX upregulated the protein expression of α-SMA in NHDFs at 72 hours; however, the magnitude and speed of the increase were less than in HCFs. MMP1 production exhibited a similar tendency (Fig 3). Meanwhile, the early response of fibroblasts, which refers to IL-6 and TGF-β induction, was equivalent in HCFs and NHDFs. These results may indicate that humoral factors do not cause the difference in response to DOX. Little is known about the difference between cardiac fibroblasts and dermal fibroblasts. Pérez-Rodríguez et al. observed that their cell migration abilities in response to cytokines are distinct [29]. Although several reports have demonstrated that trans-differentiation and MMP1 expression in dermal fibroblasts is associated with dermal wound healing and fibrosis, we revealed that the response of these cells to DOX is less than that of CFs. Lindner et al. demonstrated that the gene expression patterns in fifty fibroblasts isolated from multiple sites were highly diverse. They also noted that cell proliferation activity was the lowest in cardiac fibroblasts compared with dermal and lung fibroblasts. Moreover, they showed that the basal MMP1 level was notably higher in cardiac fibroblasts and TNFα-induced MMP1 upregulation was highest in dermal fibroblasts [30].

Myofibroblasts are known as major players in cardiac fibrosis due to their ability to secrete ECM and remodel cardiac tissue [31]. We observed that DOX increased IL-6 and TGF-β mRNA levels at 3 and 6 hours in HCFs (Fig 4A and 4B). Subsequently DOX increased α-SMA mRNA expression at 24 hours (Fig 2A) and α-SMA protein expression at 48 hours in HCFs (Fig 2B). These findings are compatible with previous reports showing that IL-6, TGF-β and PDGF are the trans-differentiation inducing factors for cardiac fibroblasts [10]. Evidence obtained from clinical trials revealed that chronic inflammatory responses are closely associated with chronic heart failure. Especially, IL-6, IL-1 and tumor necrosis factor (TNF) α are upregulated in HF patients [32]. Our results suggest that DOX raised the production of cytokines followed by trans-differentiation initiation and MMP1 and collagen production.

Several reports have supposed that elevated MMP1 (collagenase1) expression causes HF due to activation of ECM turnover and systolic dysfunction of myocardium [19] [21]. Polyakovaet et al. showed that there was a high correlation between the development of cardiac hypertrophy and MMP1 expression, and the main source of MMP1 secretion was CFs [33]. In addition, MMPs play a role not only in degradation of the ECM but also in the fibrosis process. MMPs release growth factors and matrikines that facilitate the collagen synthesis relative to its degradation [34]. MMP1 production by CFs has been found to be upregulated by anoxia-reoxygenation, angiotensin II and PDGF [35] [36] [37]. From the view point of molecular mechanisms, NF-kappa B and AP-1 activation has been shown to induce MMP1 protein expression in CFs [38]. Our data showed that DOX accelerated the mRNA, protein expression and activity of MMP1 (Fig 3). A PI3K/Akt inhibitor attenuated the production of MMP1 induced by DOX. Hence, we assumed that DOX increased MMP1 production by HCFs via PI3K/Akt signaling. DOX-induced MMP1 upregulation impairs the balance of ECM in HCFs, resulting in cardiac dysfunction and fibrosis.

It has been reported that TGF-β/Smad signaling is the canonical pathway that mediates differentiation to myofibroblasts, along with some non-canonical pathways (*i*.*e*., FAK, PI3K/Akt, JNK and p38MAPK) [25] [26]. We also previously reported that TGF-β increased trans-differentiation of HCFs and IL-6 production via the PI3K/Akt signaling pathway [18]. In this report, we demonstrated that DOX phosphorylated S6K, Akt and Smad2 in a time-dependent manner. DOX did not phosphorylate other signaling molecules, such as ERK and CREB (data not shown). Moreover, a TGF-β/Smad inhibitor prevented DOX-induced α-SMA and IL-6 protein expression. In contrast, a PI3K inhibitor prevented DOX-induced MMP1 expression and production. Thus, we speculate that DOX promotes trans-differentiation and the inflammatory response of HCFs via the TGF-β/Smad pathway and DOX increases MMP1 production via the PI3K/Akt pathway.

Although many reports have established that the mechanism of DOX-induced cardiotoxicity is cardiomyocyte cell death, the effect of DOX on cardiac fibroblasts remains unclear. Therefore, the novelty of this report is that 1) we evaluated the effect of DOX on protein (*i*.*e*., cytokines and MMPs) production by HCFs in the absence of cell death and 2) we compared the effect of DOX on human cardiac fibroblasts with human dermal fibroblasts and murine cardiac fibroblasts. In conclusion, the results of this study suggest that DOX activates the inflammatory response, activity of MMP and collagen production in HCFs at low concentrations which are not enough to decrease cell viability. Furthermore, we propose that these responses are organ- and species-specific for HCFs based on our experiments using different types of human and murine fibroblasts. Our findings have suggested that the response of HCFs for DOX starts from low concentration and that MMP1 potentially becomes alert signal in HCFs. We believe that our findings might lead to new understanding of the mechanisms and early diagnosis of DOX-induced cardiotoxicity. Future studies using animal models and clinical specimens are necessary to identify the role of MMP1 in DOX-induced cardiotoxicity.

## Supporting information

S1 Fig Cell viability of HCFs exposed to DOX. HCFs were exposed to DOX (0.1 to 0.5 μM) for 48 hours (left) and 72 hours (right), and cell viability was measured with an XTT assay (n = 4; **p < 0.01, and ***p<0.001; ns: no significant difference).
(TIF)

S2 Fig A. Original image of α-SMA expression in HCFs and NHDFs exposed to DOX (0.1 μM). B. Original image of MMP1 expression in HCFs and NHDF exposed to DOX (0.1 μM) (data shown in Figs 2 and 3).
(TIF)

S3 Fig NHDFs were capable of trans-differentiation to myofibroblasts. Protein expression of α-SMA in NHDFs with or without TGF-β1 treatment (1 ng/ml) for 48 hours.
(TIF)

S4 Fig DOX enhanced protein expression of some markers presenting the trans-differentiation of HCFs. Protein expression of fibronectin, vinculin and paxillin in HCFs and NHDFs with DOX (0.1 μM).
(TIF)

S5 Fig DOX did not increase MMP2 and MMP9 mRNA expression. mRNA expression of MMP2 (left) and MMP9 (right) in the presence of DOX (0.1 μM) for 24 hours in HCFs (n = 4; ns: no significant difference).
(TIF)

S6 Fig DOX increased production of MMP1 in supernatant from HCFs. Production of MMP1 in supernatant from HCFs (left) and NHDFs (right) exposed to DOX (0.1 μM) for 48 hours (n = 4–6; *p<0.05; ns: no significant difference).
(TIF)

S7 Fig DOX increased the expression of col1a1 mRNA in HCFs but not in NHDFs. The expression of col1a1 mRNA in HCFs with DOX treatment (0.1 μM) for 24 to 72 hours and col1a1 mRNA expression in NHDFs with DOX treatment (0.1 μM) for 24 (n = 4–7; **p<0.01; ns: no significant difference).
(TIF)

S8 Fig Original image of p-S6K (T), p-Smad2, p-Akt(S), S6K, Smad2, Akt and GAPDH in HCFs exposed to DOX (0.1 μM) (data shown in Fig 5A). (TIF)

S9 Fig Original image of MMP1, α-SMA and GAPDH in HCFs exposed to DOX (0.1 μM) with LY294002 or SB431512 (data shown in Fig 5B). (TIF)

S10 Fig Original image of Fibronectin, Vinculin, Paxillin, α-SMA and GAPDH in HCFs and NHDF exposed to DOX (0.1 μM). (TIF)

S1 File Original data (data shown in Fig 1). (XLSX)

S2 File Original data (data shown in Fig 2). (XLSX)

S3 File Original data (data shown in Fig 3). (XLSX)

S4 File Original data (data shown in Fig 4). (XLSX)

S5 File Original data (data shown in Fig 5). (XLSX)

S6 File Original data (data shown in S7 Fig). (XLSX)
