# Supplementary figures and images for: Doxorubicin induces trans-differentiation and MMP1 expression in cardiac fibroblasts via cell death-independent pathways
Source: PLoS One. 2019 Sep 12;14(9):e0221940. doi: 10.1371/journal.pone.0221940 (PMC6742217; doi:10.1371/journal.pone.0221940)

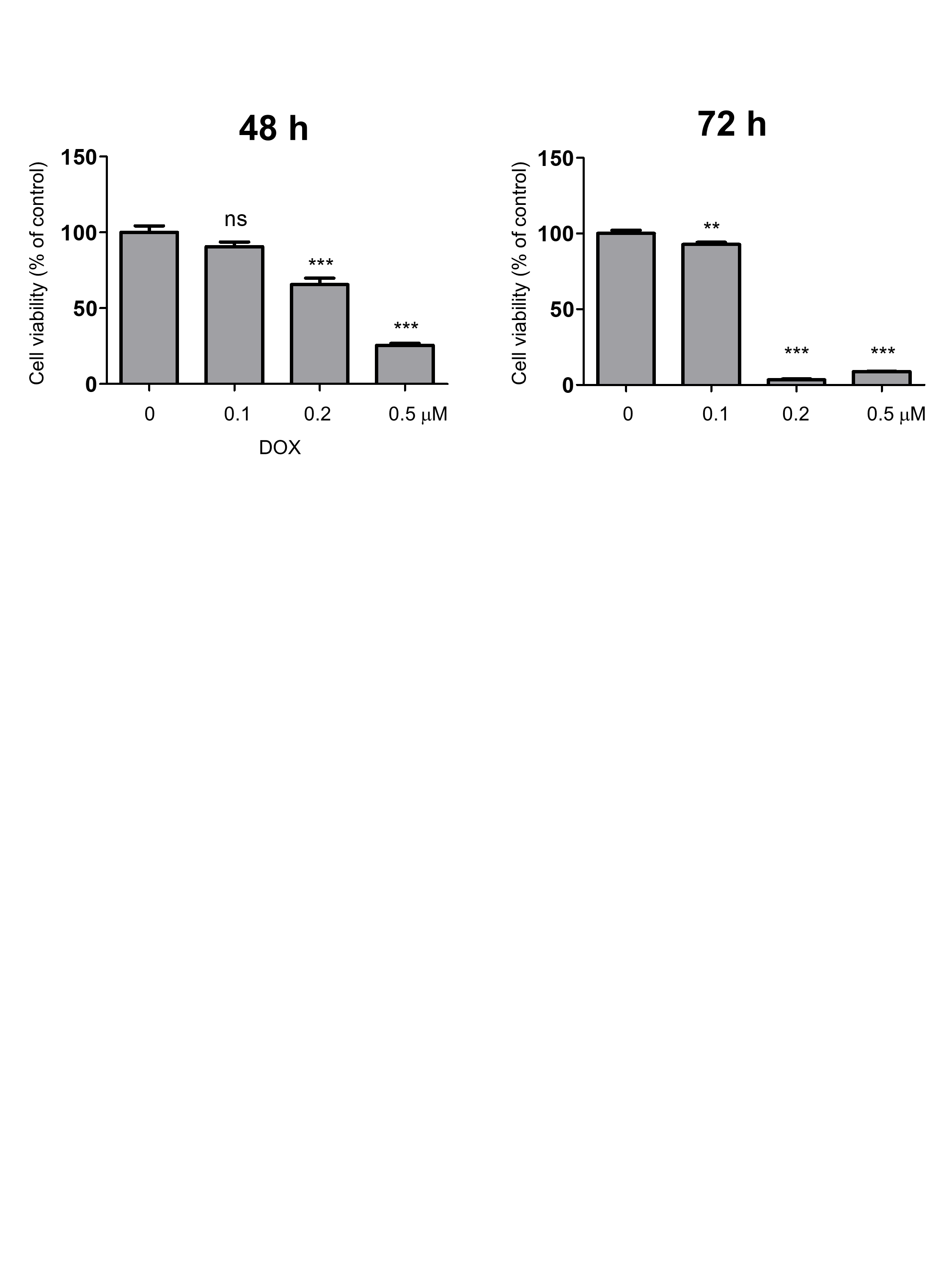

Supplement: S1 Fig — HCFs were exposed to DOX (0.1 to 0.5 μM) for 48 hours (left) and 72 hours (right), and cell viability was measured with an XTT assay (n = 4; **p < 0.01, and ***p<0.001; ns: no significant difference). (TIF) [file pone.0221940.s001.TIF]

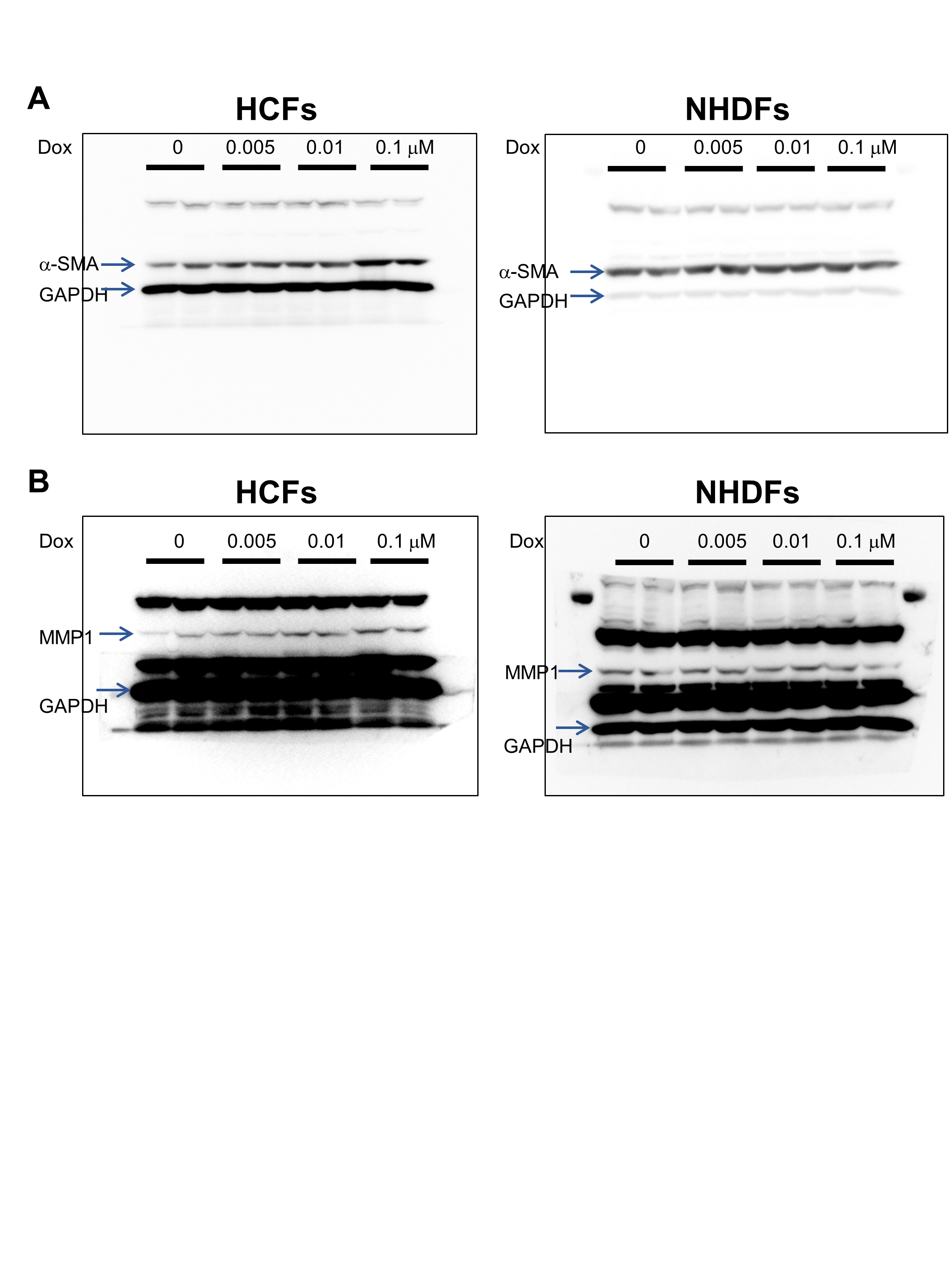

Supplement: S2 Fig — A. Original image of α-SMA expression in HCFs and NHDFs exposed to DOX (0.1 μM). B. Original image of MMP1 expression in HCFs and NHDF exposed to DOX (0.1 μM) (data shown in Figs 2 and 3). (TIF) [file pone.0221940.s002.TIF]

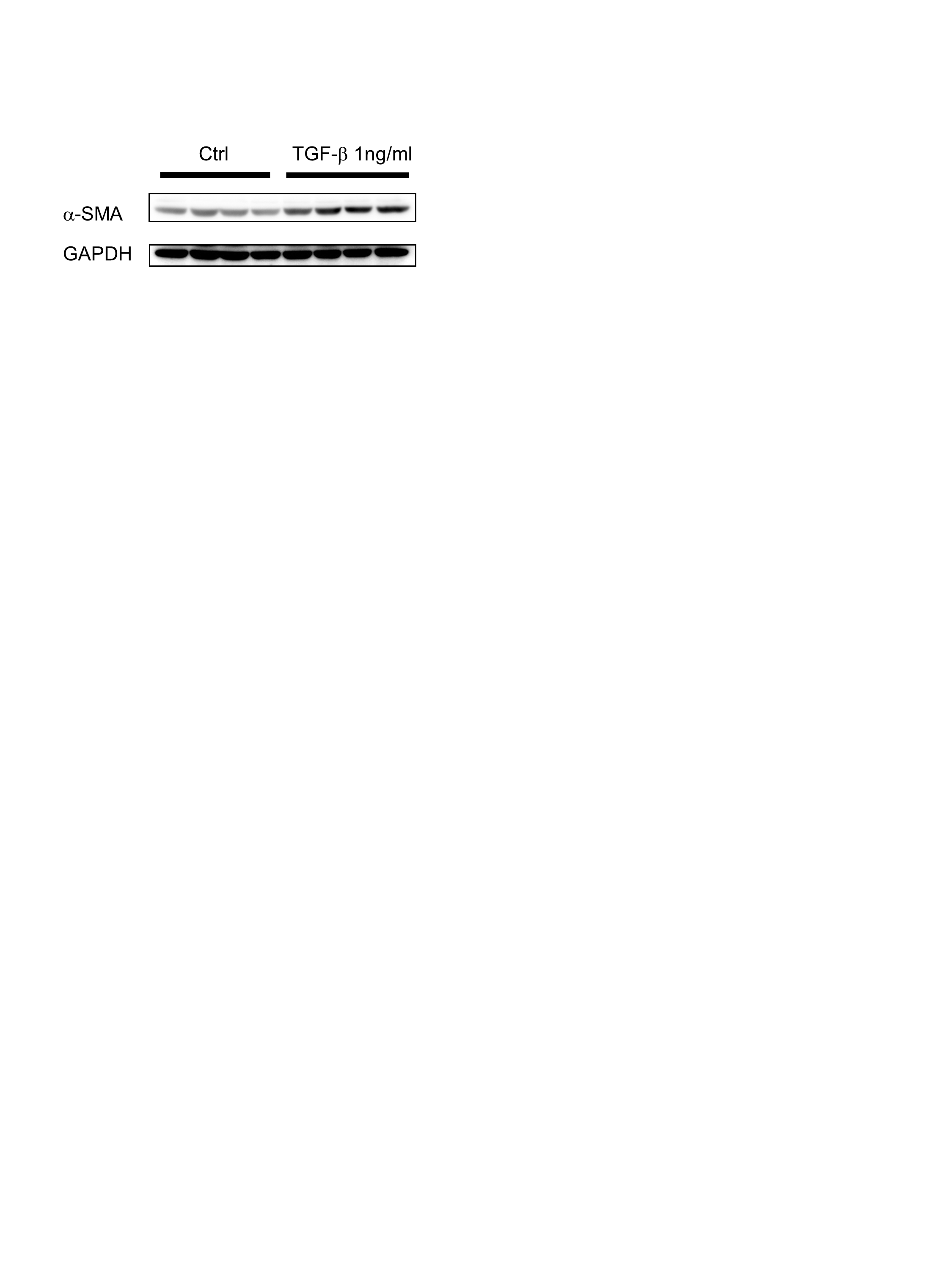

Supplement: S3 Fig — Protein expression of α-SMA in NHDFs with or without TGF-β1 treatment (1 ng/ml) for 48 hours. (TIF) [file pone.0221940.s003.TIF]

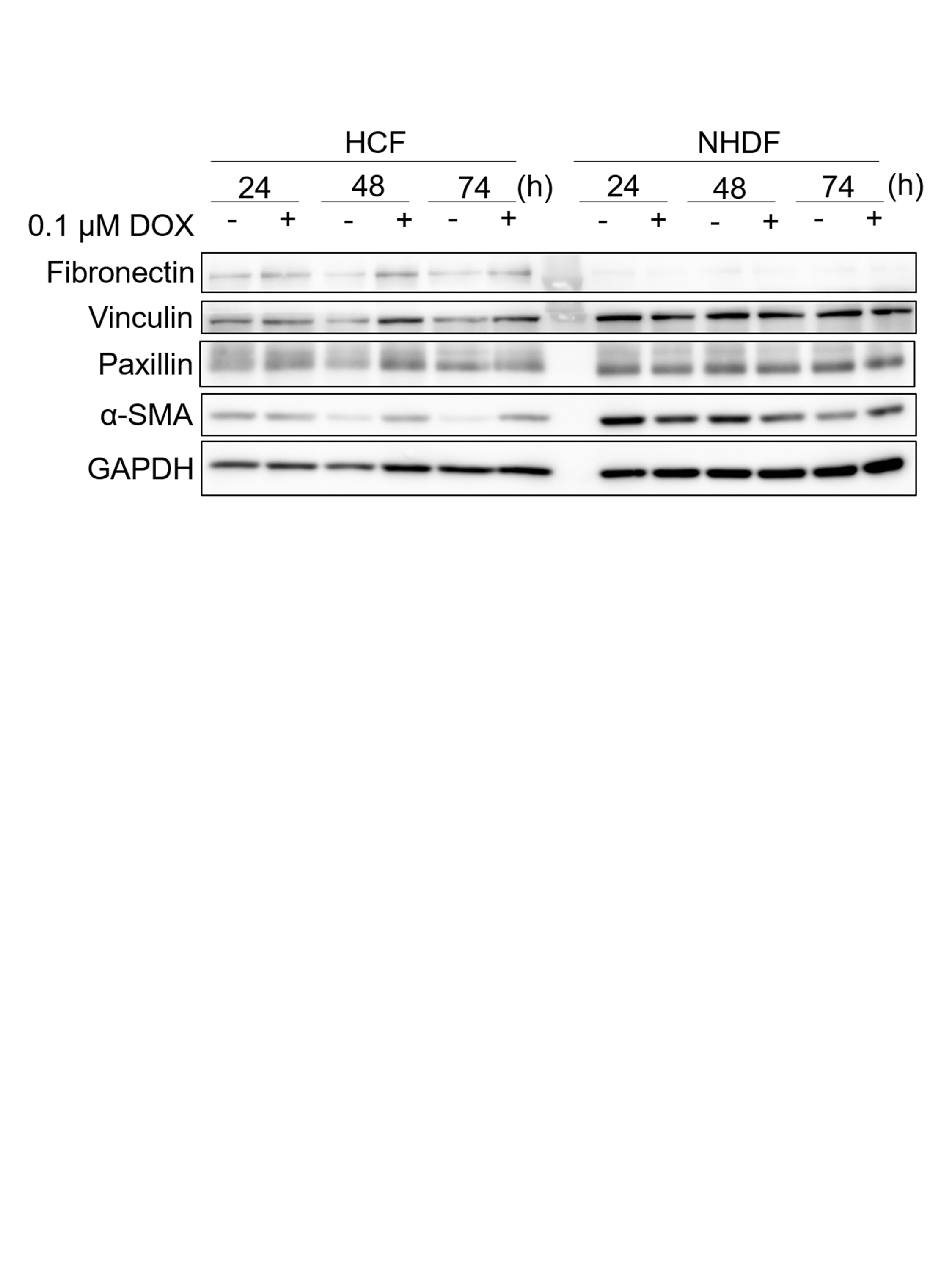

Supplement: S4 Fig — Protein expression of fibronectin, vinculin and paxillin in HCFs and NHDFs with DOX (0.1 μM). (TIF) [file pone.0221940.s004.TIF]

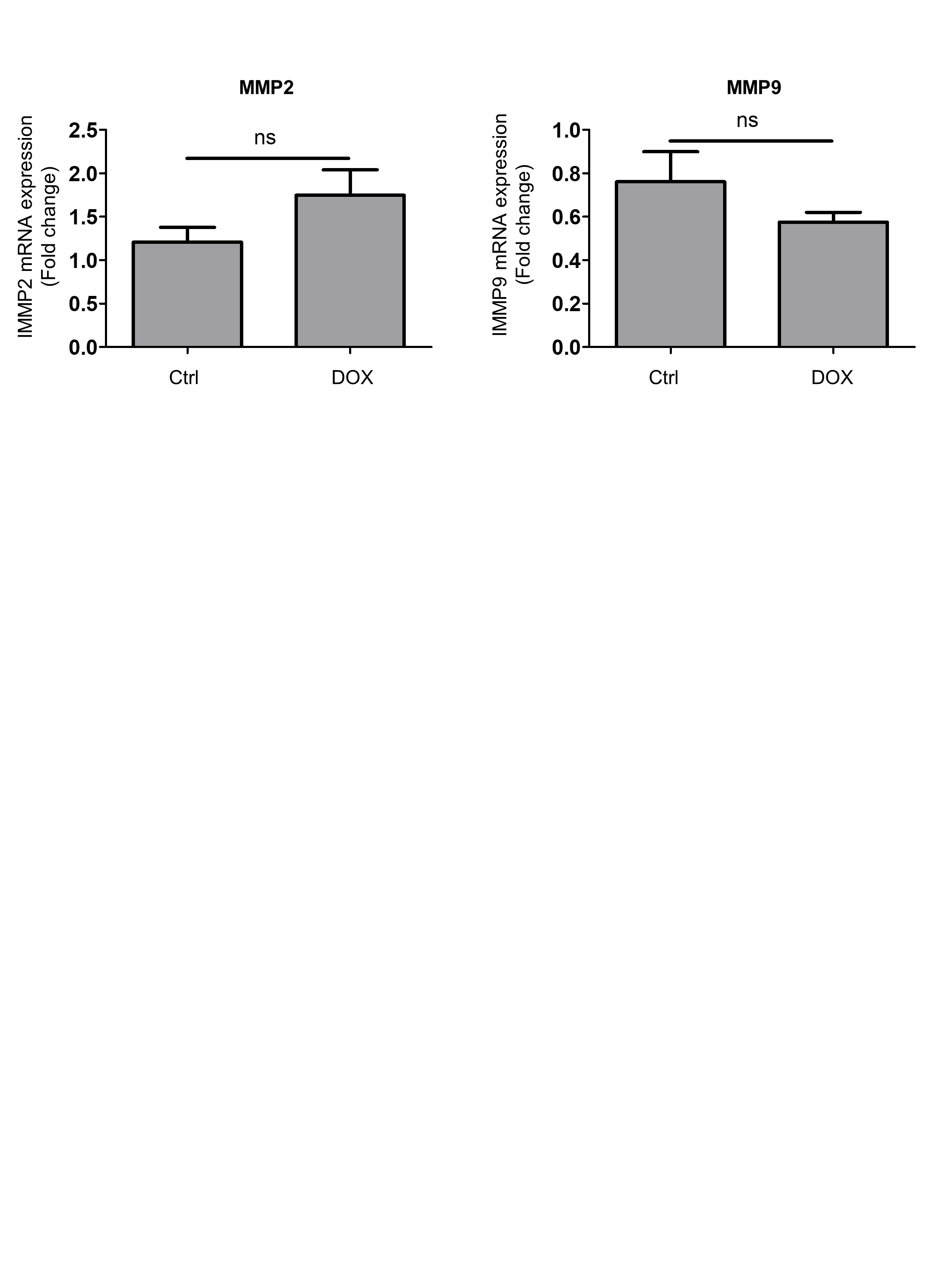

Supplement: S5 Fig — mRNA expression of MMP2 (left) and MMP9 (right) in the presence of DOX (0.1 μM) for 24 hours in HCFs (n = 4; ns: no significant difference). (TIF) [file pone.0221940.s005.TIF]

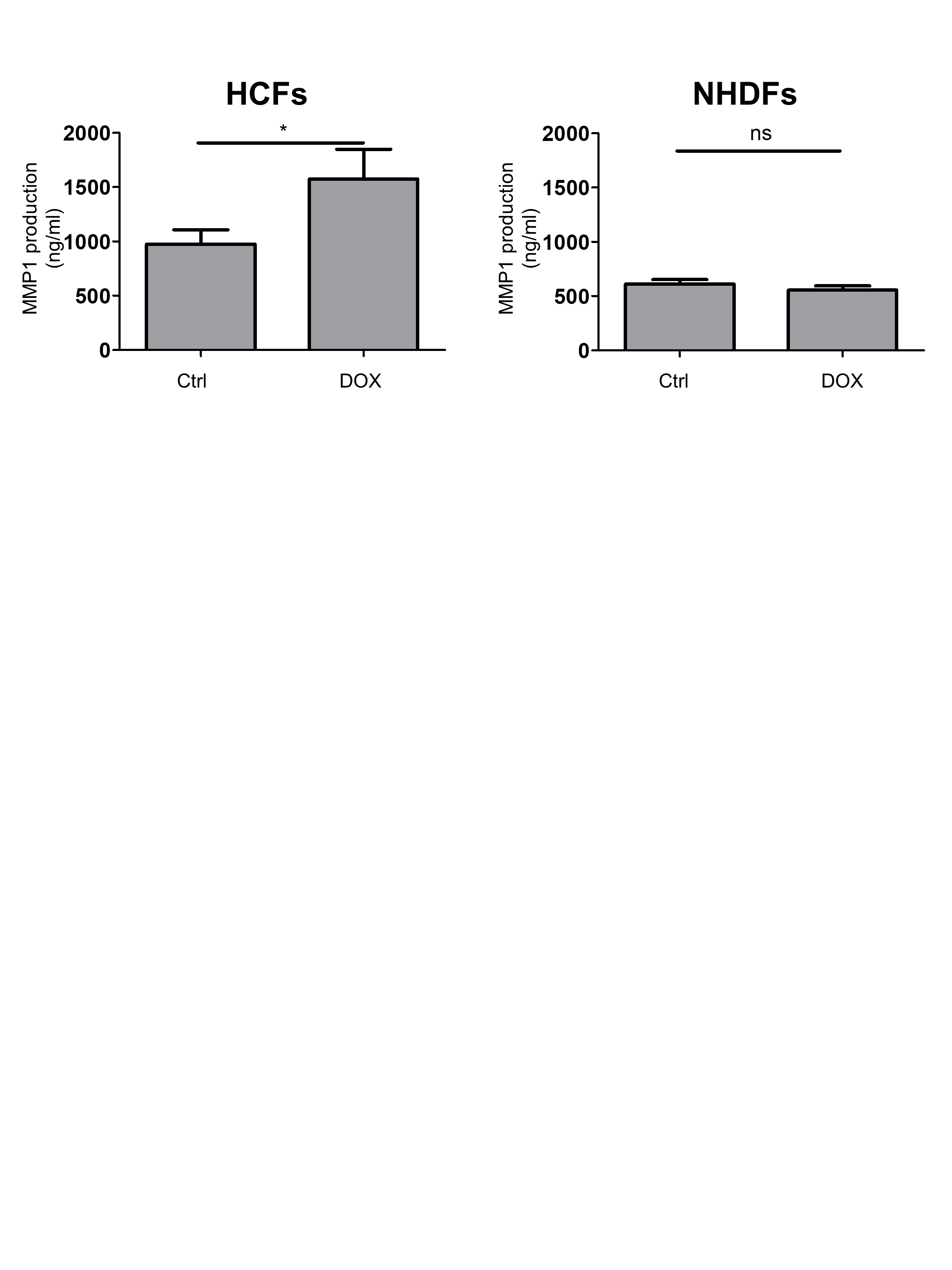

Supplement: S6 Fig — Production of MMP1 in supernatant from HCFs (left) and NHDFs (right) exposed to DOX (0.1 μM) for 48 hours (n = 4–6; *p<0.05; ns: no significant difference). (TIF) [file pone.0221940.s006.TIF]

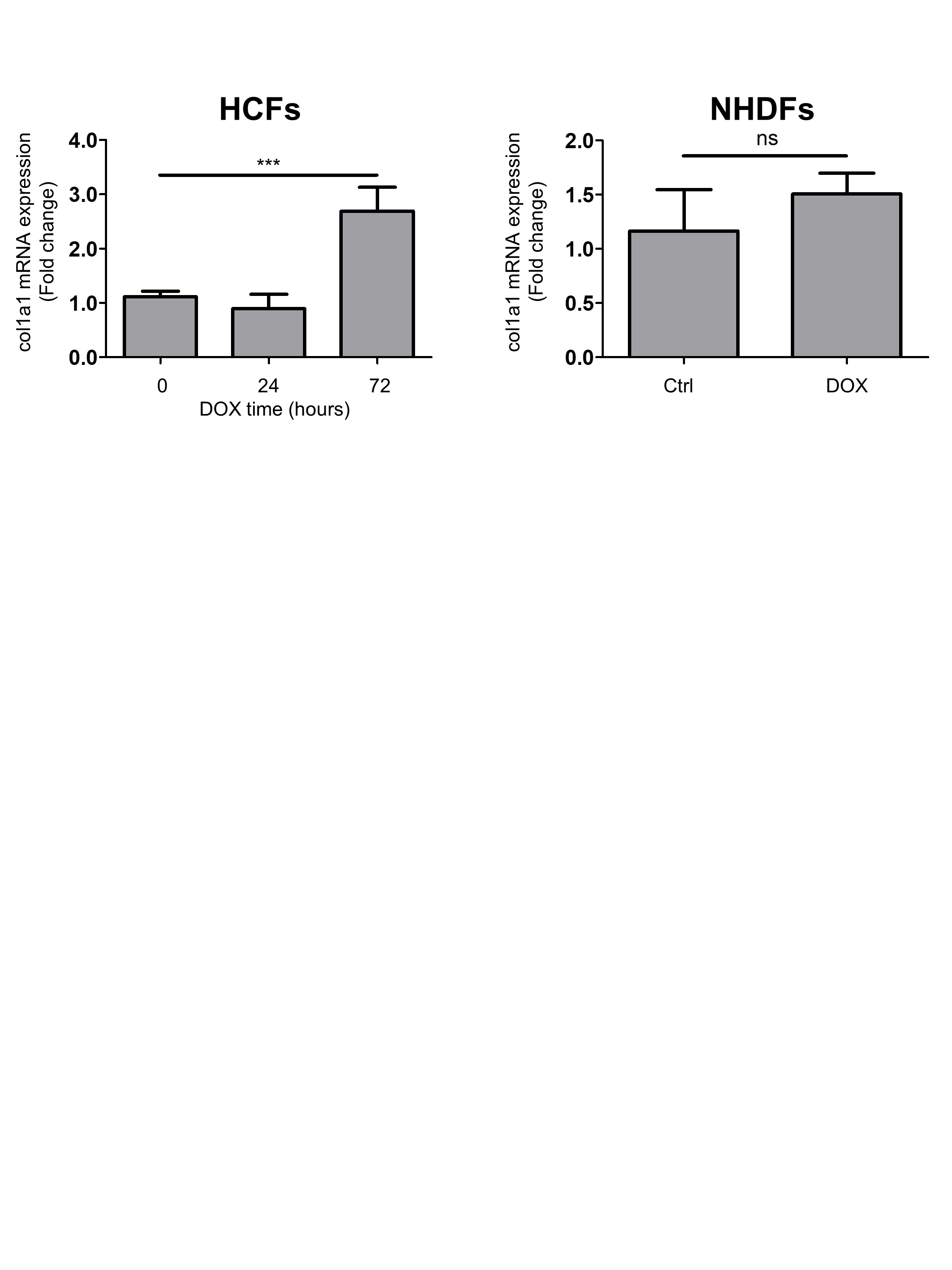

Supplement: S7 Fig — The expression of col1a1 mRNA in HCFs with DOX treatment (0.1 μM) for 24 to 72 hours and col1a1 mRNA expression in NHDFs with DOX treatment (0.1 μM) for 24 (n = 4–7; **p<0.01; ns: no significant difference). (TIF) [file pone.0221940.s007.TIF]

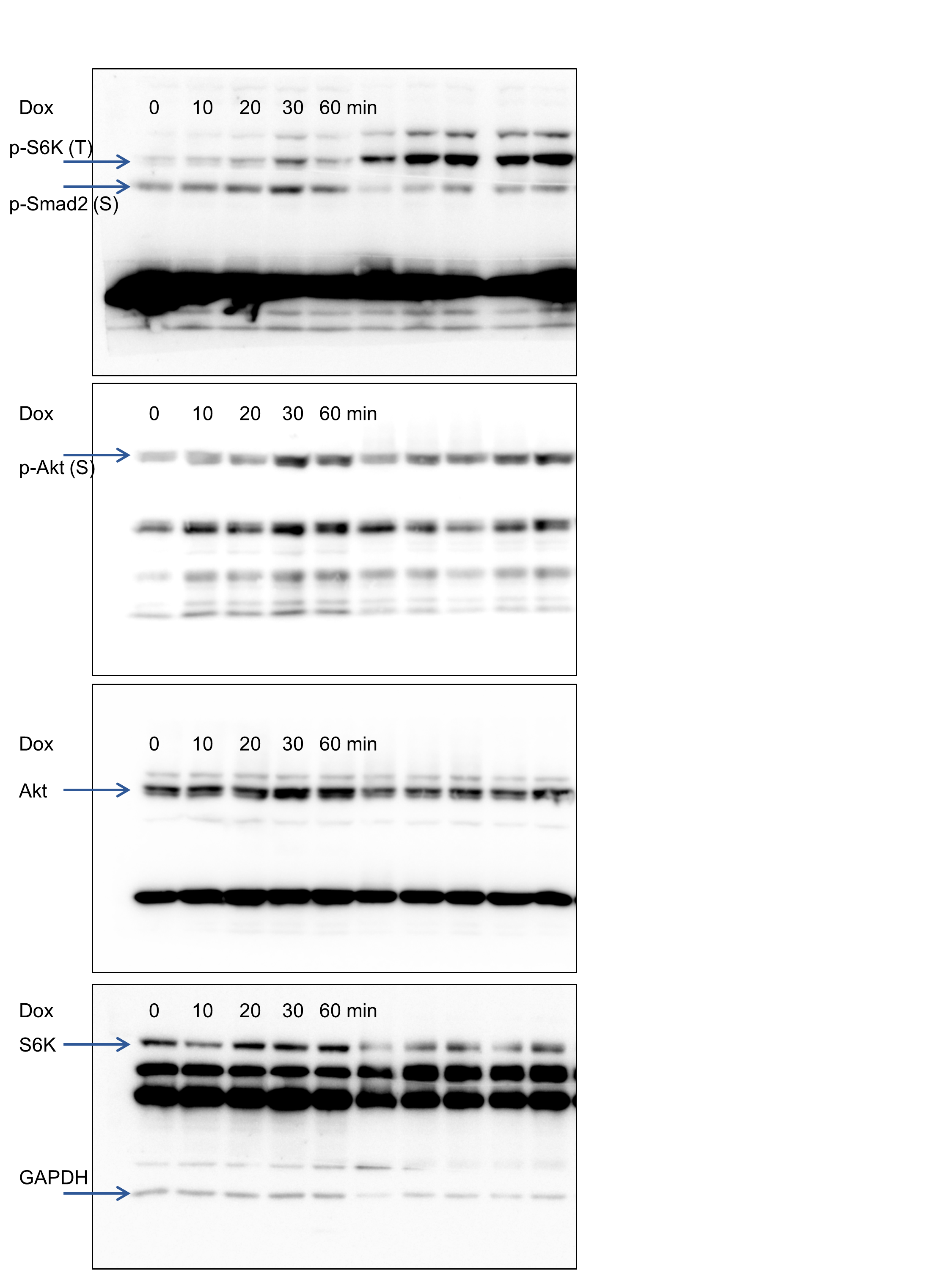

Supplement: S8 Fig — (TIF) [file pone.0221940.s008.TIF]

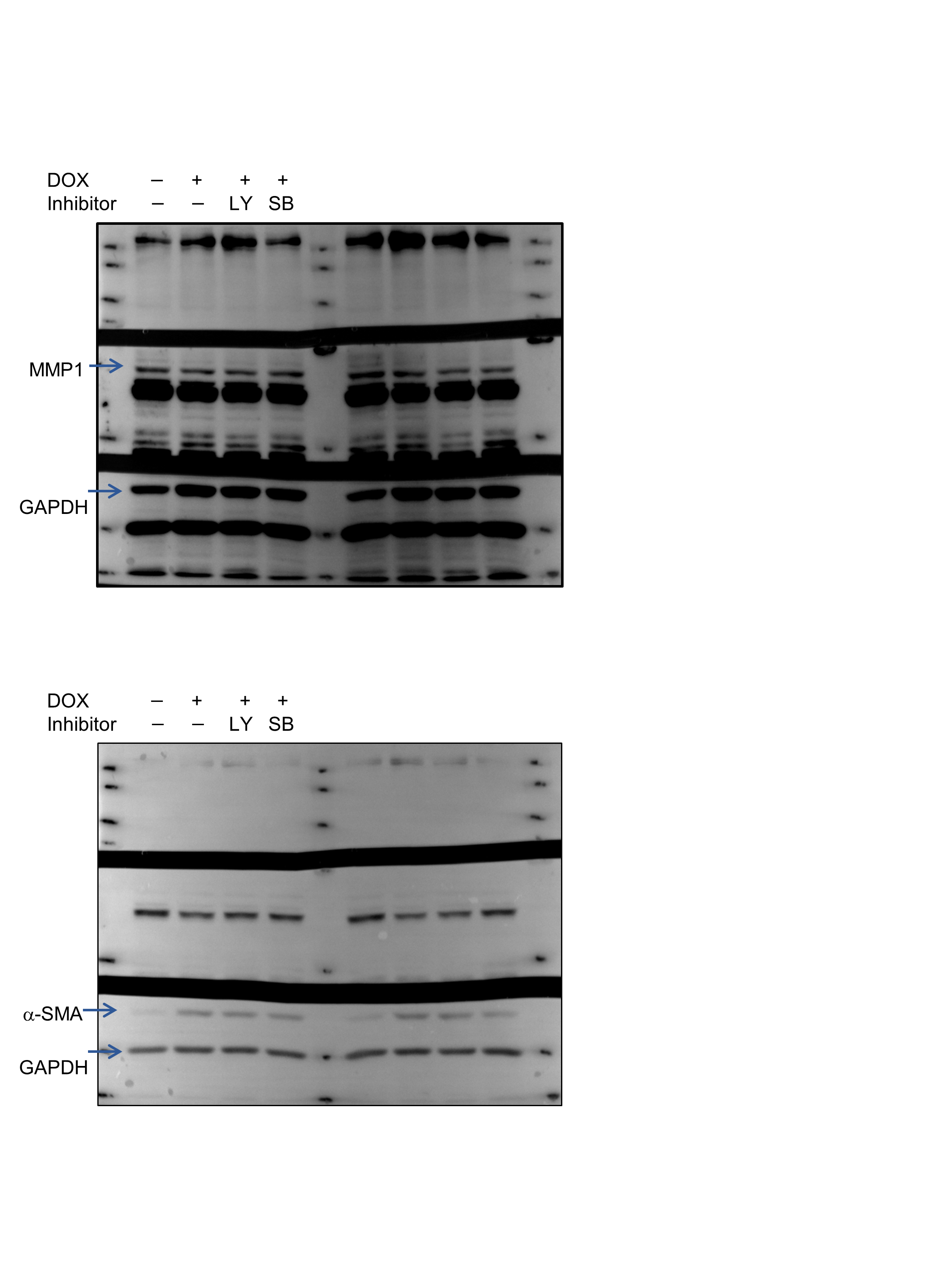

Supplement: S9 Fig — (TIF) [file pone.0221940.s009.TIF]

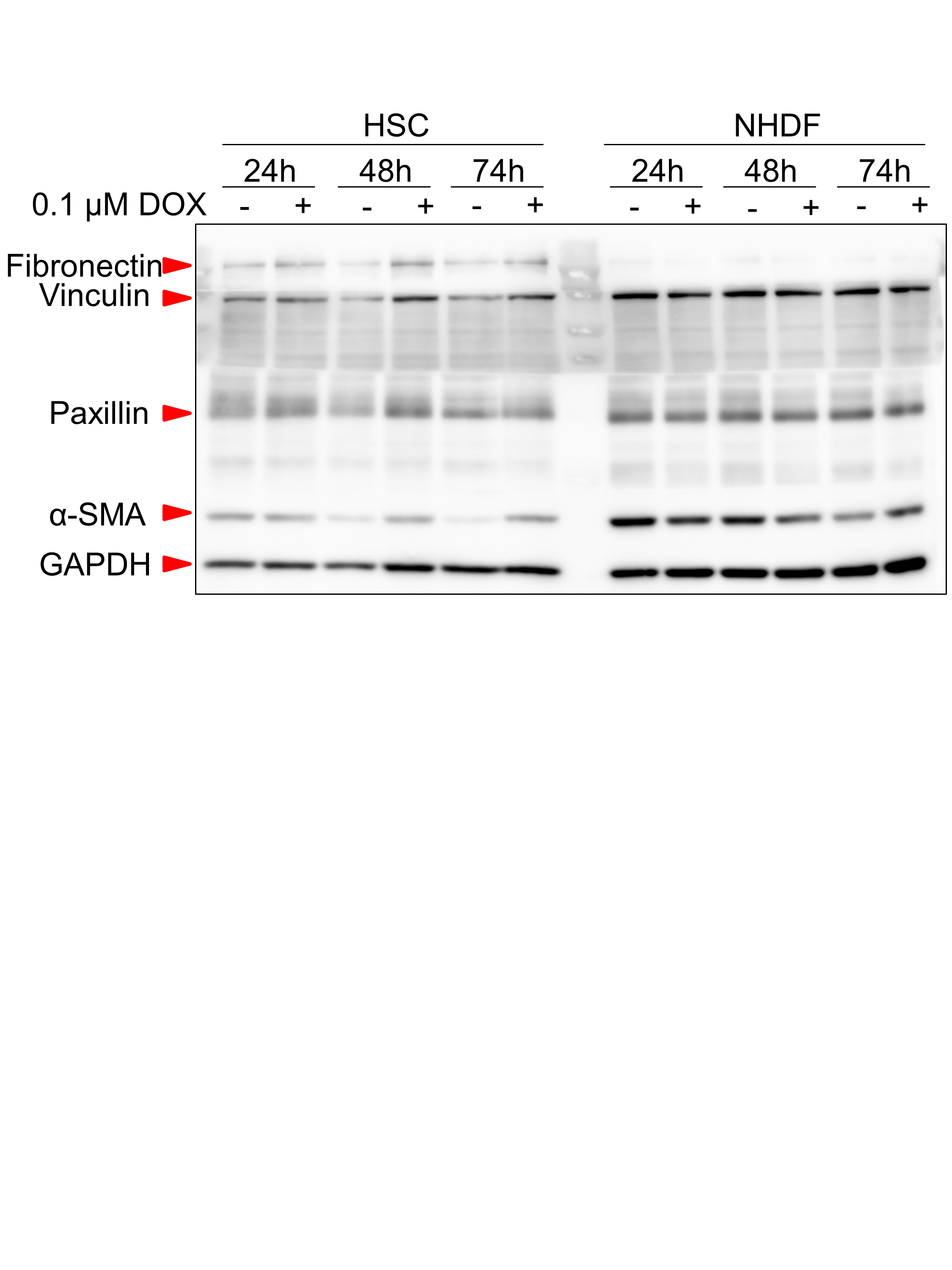

Supplement: S10 Fig — (TIF) [file pone.0221940.s010.tif]
